# Influence of 4 weeks of downhill running on calcium sensitivity of rat single muscle fibers

**DOI:** 10.14814/phy2.15450

**Published:** 2022-10-12

**Authors:** Emma F. Hubbard, Avery Hinks, Parastoo Mashouri, Geoffrey A. Power

**Affiliations:** ^1^ Department of Human Health and Nutritional Sciences, College of Biological Sciences University of Guelph Guelph Ontario Canada

**Keywords:** calcium sensitivity, eccentric, KTR, rate of force development, resistance training, rodent, running

## Abstract

Improved Ca^2+^ sensitivity has been suggested as a mechanism behind enhancements in muscle mechanical function following eccentric training. However, little is known regarding the effects of eccentric training on single muscle fiber Ca^2+^ sensitivity. Adult male Sprague–Dawley rats (sacrificial age ~18 weeks; mass = 400.1 ± 34.8 g) were assigned to an eccentric training (*n* = 5) or sedentary control group (*n* = 6). Eccentric training consisted of 4 weeks of weighted downhill running 3×/week at a 15° decline and 16 m/min for 35 min per day in 5‐min bouts. After sacrifice, vastus intermedius single muscle fibers were dissected, chemically permeabilized, and stored until testing. Fibers (*n* = 63) were isolated, and standard Ca^2+^ sensitivity, force, rate of force redevelopment (*k*_tr_), and active instantaneous stiffness tests were performed using [Ca^2+^] ranging from 7.0 to 4.5. Following all mechanical testing, fiber type was determined using SDS‐PAGE. There was no difference in pCa_50_ (i.e., [Ca^2+^] needed to elicit half of maximal force) between groups or between fiber types. However, when comparing normalized force across pCa values, fibers from the control group produced greater forces than fibers from the trained group at lower Ca^2+^ concentrations (*p* < 0.05), and this was most evident for Type I fibers (*p* = 0.002). Type II fibers produced faster (*p* < 0.001) *k*_tr_ than Type I fibers, but there were no differences in absolute force, normalized force, or other measures of mechanical function between fibers from the trained and control groups. These findings indicate that eccentric training does not appear to improve single muscle fiber Ca^2+^ sensitivity.

## Graphical abstract

Little is known about the effects of eccentric training on Ca^2+^ sensitivity in single muscle fibers. 4 weeks of weighted downhill running produced minimal effects on mechanical function of single fibers from rat vastus intermedius. Changes to Ca^2+^ sensitivity are unlikely to contribute to improvements in mechanical function following eccentric training.

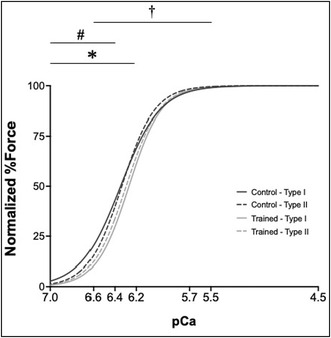

## 1 INTRODUCTION

Eccentric (i.e., active muscle lengthening) training has been shown to improve muscle mechanical function (Douglas et al., 2017; Franchi et al., 2014). A systematic review concluded that eccentric training increases force production in subsequent acute concentric, isometric, and eccentric contractions and that the capacity for eccentric training to improve mechanical function is greater than that of other training modalities (Douglas et al., 2017). At the whole muscle level, eccentric training has been shown to increase muscle size and strength and improve neural activation with lower oxygen consumption required for activation (Higbie et al., 1996; Kaminski et al., 1998; Lastayo et al., 1999). Furthermore, architectural and strength adaptations seen following eccentric training appear to be mechanistically different from responses to concentric training (Franchi et al., 2014). However, despite an abundance of evidence for the role of eccentric exercise to improve muscle mechanical function, an understanding of the underlying cellular mechanisms of these training outcomes is lacking.

The underlying mechanism of increased force production following eccentric training is likely a combination of neural, morphological, and architectural factors (Douglas et al., 2017). Additionally, it has been speculated that improved Ca^2+^ handling may play a role in performance enhancement following eccentric training. Increases in maximal isometric force production and reductions in low‐frequency fatigue observed following a 6‐week high speed eccentric training protocol in rat plantar flexors indicate that this adaptation may be related to improved Ca^2+^ sensitivity (Willems & Stauber, 2002). Presently, few studies have investigated the effects of eccentric training on Ca^2+^ sensitivity of single muscle fibers. Following eccentric training in adjuvant‐induced arthritis rats who have impaired Ca^2+^ regulation of force, single‐fiber Ca^2+^‐activated force was restored almost to the level of healthy controls (Himori et al., 2019). However, it was not reported whether Ca^2+^ sensitivity improved. Another study examined the ability of low‐intensity eccentric training to rehabilitate skeletal muscle following disuse in *mdx* mice (Pedrazzani et al., 2021). Active force improved in trained compared to sedentary *mdx* mice, but Ca^2+^ sensitivity was not impaired in *mdx* fibers compared to wild‐type fibers; thus, the effect of training on Ca^2+^ sensitivity was not assessed (Pedrazzani et al., 2021). The absence of Ca^2+^ sensitivity data in these works illustrates a clear gap in the literature regarding the effect of eccentric training on muscle contractility.

While no differences in Ca^2+^ sensitivity have been observed between endurance‐trained athletes and age‐matched controls (Fitts et al., 1989; Widrick et al., 1996), resistance‐based exercise in humans has been shown to increase Ca^2+^ sensitivity, particularly in Type I muscle fibers (Godard et al., 2002; Malisoux et al., 2006). Accordingly, muscle unloading has been associated with lower Type I fiber Ca^2+^ sensitivity in both humans and rodents (McDonald & Fitts, 1995; Widrick et al., 1998). However, not all training types have been successful at modulating Ca^2+^ sensitivity, and high‐intensity interval training programs in humans have even resulted in decreases in Ca^2+^ sensitivity in Type I fibers (Lamboley et al., 2020). Interestingly, reversible oxidation‐related increases in Type II fiber Ca^2+^ sensitivity have been observed following training and are thought to counteract any metabolite‐induced Ca^2+^ sensitivity reduction, but these increases are almost completely lost when treated with a reducing agent or after 24 h of rest (Lamboley et al., 2020; Xu et al., 2018). With respect to eccentric training in particular, despite the lack of Ca^2+^ sensitivity data in previous work, it is known that Ca^2+^ sensitivity increases as a function of sarcomere length (Joumaa & Herzog, 2014; Martyn & Gordon, 2001). Data from our lab showed an increase in the number of serial sarcomeres and resting sarcomere length following eccentric training (i.e., weighted downhill running) in the rat soleus (Hinks et al., 2022). Therefore, it is possible that following chronic eccentric training, sarcomeres may be operating at a longer length, resulting in a corresponding increase in Ca^2+^ sensitivity.

The purpose of this study was to examine Ca^2+^ sensitivity and mechanical function of rat vastus intermedius single muscle fibers following 4 weeks of weighted downhill running (i.e., eccentric) training. It was hypothesized that weighted downhill running would improve mechanical function, and Ca^2+^ sensitivity would be greater in trained compared to control rats, particularly in Type I fibers.

## 2 MATERIALS AND METHODS

### 2.1 Animals

Eleven adult male CD® Sprague–Dawley rats (sacrificial age ~18 weeks; mass = 400.1 ± 34.8 g) were obtained (Charles River Laboratories) with approval from the University of Guelph's Animal Care Committee and all protocols following CCAC guidelines. Rats were housed at 23°C in groups of three and given ad libitum access to a Teklad global 18% protein rodent diet (Envigo, Huntington) and room‐temperature water. Following 1 week of acclimation to housing conditions and familiarization with the vests and treadmills, rats were assigned to one of two experimental groups: eccentric training (i.e. weighted downhill running) and sedentary control. After 4 weeks of training, rats recovered for 72 h before sacrifice by CO_2_ asphyxiation followed by cervical dislocation before experimental testing. There was no difference in body mass between groups.

### 2.2 Training protocol

Two weeks prior to training, rats were handled for 1 h on 3 consecutive days to reduce their stress levels when later applying the vests. The next week (i.e., 1 week before training), the rats underwent 5 consecutive days of familiarization sessions on the treadmill, each consisting of three 3‐min sets at a 0° grade and 10–12 m/min speed, with 2 min of rest between each set. In the first two familiarization sessions they did not wear vests, in the third session they wore an unweighted vest for 1/3 sets, in the fourth session they wore an unweighted vest for 2/3 sets, and in the fifth session they wore an unweighted vest for all 3 sets. Rats who were not compliant to the treadmill after 3 days of attempted familiarization were made controls instead.

The training protocol consisted of 4 weeks of downhill running training 3 days/week, with custom‐made weighted vests (Hinks et al., 2022) to add load during running. Rats ran on an EXER 3/6 animal treadmill (Columbus Instruments) at a 15° decline. Each session began at a speed of 10 m/min, which was increased by 1 m/min up to the 16 m/min target (Chen et al., 2020). To enhance eccentric loading during the downhill running, load was added via weighted vests up to 15% of body mass. During the first week of training, the added load on the vest was 5% of body mass and rats completed 3 × 5‐min bouts on the first day of training, 5 × 5‐min bouts on the second day, and 7 × 5‐min bouts on the third day. During the second week of training, rats continued to complete 7 × 5‐min bouts and weight was increased to 10% of body mass. Weight was further increased to 15% of body mass during the third week while the number of bouts remained unchanged. In the fourth week, the weight was re‐adjusted to 15% of the new body mass with the same number of bouts. Rats were given 2 min of rest after each 5‐min bout. Each training session took place at approximately the same time of day (between 9 and 11 a.m.).

### 2.3 Solutions

The dissecting solution was composed of the following (in mM): K‐proprionate (250), Imidazole (40), EGTA (10), MgCl_2_·6H_2_O (4), and Na_2_H_2_ATP (2), H_2_O. The storage solution was composed of K‐proprionate (250), Imidazole (40), EGTA (10), MgCl_2_·6H_2_O (4), Na_2_H_2_ATP (2), glycerol (50% of total volume after transfer to 50:50 dissecting: glycerol solution), as well as leupeptin (Sigma) protease inhibitors. The skinning solution with Brij 58 was composed of K‐propionate (250), Imidazole (40), EGTA (10), MgCl_2_·6H_2_O (4), 1 g of Brij 58 (0.5% w/v). The relaxing solution was composed of Imidazole (59.4), K.MSA (86), Ca(MSA)_2_ (0.13), Mg(MSA)_2_ (10.8), K3EGTA (5.5), KH_2_PO_4_ (1), H_2_O, Leupeptin (0.05), Na_2_ATP (5.1), as well as leupeptin (Sigma) protease inhibitors. The preactivating solution consisted of KPr (185), MOPS (20), Mg(CH_3_COOH)_2_ (2.5), and ATP (2.5). The activating solution consisted of Ca^2+^, Mg, EGTA (15), MOPS (80), ATP (5), CP (15), K (43.27), Na, and H_2_O. Ca^2+^, Mg, and Na concentrations were specific to the pCa level. These concentrations are listed below:

1. 4.5: Ca^2+^ (14.87), Mg (6.93), Na (13.23)
2. 5.5: Ca^2+^ (12.83), Mg (6.97), Na (13.4)
3. 5.7: Ca^2+^ (11.83), Mg (7), Na (13.4)
4. 6.2: Ca^2+^ (8.1), Mg (7.07), Na (13.4)
5. 6.4: Ca^2+^ (6.4), Mg (7.07), Na (13.4)
6. 6.6: Ca^2+^ (4.8), Mg (7.1), Na (13.4)
7. 7.0: Ca^2+^ (2.37), Mg (7.17), Na (13.4)

All solutions were adjusted to a pH of 7.0 with the appropriate acid (HCl) or base (KOH).

### 2.4 Tissue preparation

Rats were sacrificed 3 days after their final training session. Vastus intermedius muscles were harvested from the rats following sacrifice and were submerged in chilled dissecting solution. The vastus intermedius was selected as the hindlimb muscles of rats undergo active lengthening (i.e., eccentric contractions) during similar downhill running protocols as the current study (Butterfield et al., 1985; Lynn & Morgan, 1994). Importantly, the vastus intermedius has repeatedly been shown to have strong adaptations to downhill eccentric training in the form of increased serial sarcomere number (Butterfield et al., 1985; Hinks et al., 2022; Lynn & Morgan, 1994). While in the dissecting solution, several muscle fiber bundles of approximately 0.5–1 mm in width and 3 mm in length were dissected and transferred to a tube containing 2.5 ml of chilled skinning solution on ice for 30 min to be chemically permeabilized (Mazara et al., 2021). Gentle agitation was performed to ensure that all bundles were constantly submerged in the solution, resulting in equal permeabilization. The bundles were then washed with fresh chilled dissecting solution, gently agitated to remove any remaining skinning solution, and stored in a separate tube containing fresh storage solution. The bundles were incubated for 24 h at 4°C. 0.6 ml tubes were prepared with fresh storage solution, and the bundles were placed separately in each tube and placed in a freezer at −80°C until mechanical testing began (Mashouri et al., 2021).

### 2.5 Mechanical testing and force measurements

On the day of mechanical testing, the tubes containing the muscle bundles were removed from the −80°C freezer and placed in a chilled relaxing solution on ice where they remained for the duration of the testing protocol. Single fibers were dissected from the bundles in relaxing solution, then transferred into a temperature‐controlled chamber (15°C) filled with relaxing solution and tied with nylon suture knots between a force transducer (model 403A; Aurora Scientific) and a length controller (model 322C; Aurora Scientific). The average sarcomere length (SL) was measured using a high‐speed camera (Aurora Scientific). Before starting the testing protocol, a “fitness” contraction was performed at a SL of 2.5 μm (Burkholder & Lieber, 2001; Ledvina & Segal, 1995; Smith et al., 2021) in pCa 4.5 to ensure the ties were not loose and the fiber did not have extreme SL nonuniformity caused by accidental damage during the single muscle fiber dissection step or the fiber tying process. After the fitness test, SL was re‐measured and, if necessary, re‐adjusted to 2.5 μm. Fiber length (L_0_) was recorded, and fiber diameter was measured at three different points along the fiber using a reticule on the microscope. The average of the fiber diameters was used to calculate cross‐sectional area (CSA) assuming circularity. To initiate activation, the fibers were transferred to a pre‐activating solution (reduced Ca^2+^ buffering capacity with ATP) for 20 s, then to an activating solution (pCa 7.0, 6.6, 6.4, 6.2, 5.7, 5.5, and 4.5) for 30 s (Mashouri et al., 2021; Mazara et al., 2021). Single fiber optimal force (*P*_o_) at each pCa level was taken as the highest 500 ms moving average along the force‐time curve during the 30 s window.

Once force reached a plateau, a length step was induced to measure rate of force redevelopment at each pCa (*k*_tr_; Brenner & Eisenberg, 1986). This was performed by rapidly shortening the fiber with a ramp of 10 L_o_/s by 15% of L_o_ and then a rapid (500 L_o_/s) re‐stretch back to L_o_. At saturating Ca^2+^ levels, the rapid shortening causes all cross‐bridges to break, and the re‐stretch allows further dissociation of any remaining cross‐bridges followed by a redevelopment of force, independent of Ca^2+^‐dependent regulatory proteins at L_o_. A monoexponential equation, *y* = *a* (1 − e^−*kt*^) + *b*, was fit to the redevelopment curve to determine *k*_tr_.

To assess the proportion of attached cross‐bridges, active instantaneous stiffness (*k*) tests were performed. Instantaneous stiffness was assessed by inducing a rapid (500 L_o_/s) stretch of 0.3% of L_o_ and dividing the change in force during the stretch by the length step.

A final contraction was performed in pCa 4.5 to assess % force drop over the course of testing to ensure that no damage sustained by the fiber during contractions would affect results.

### 2.6 Fiber exclusion

*n* = 36 control fibers and *n* = 37 trained fibers were tested. Fibers with a force drop >26% were excluded from analysis. Based on this criteria, *n* = 5 control fibers (Type I, 0; Type II, 5) and *n* = 5 trained fibers (Type I, 2; Type II, 3) were excluded. Any fiber that slipped or ripped before the completion of testing at all pCa levels was removed from the testing apparatus and excluded from the data set. The fibers that remained untested at the end of the day were discarded, and a new bundle was selected from the freezer on each subsequent mechanical testing day. Testing days alternated between control and training groups to minimize any possible storage effect. Additionally, data points for any measure that were more than 3 SD away from the mean were excluded for analysis.

### 2.7 Fiber type

After the completion of mechanical testing, the fiber was placed in 15 μl of solubilization buffer containing 61 mM tris (pH 6.8), 11% (v/v) glycerol, 2.78% (w/v) sodium dodecyl sulfate (SDS), 5% 2‐β mercaptoethanol, and 0.02% (w/v) bromophenol blue and stored at −20°C until fiber typing was to begin (Mashouri et al., 2021; Mazara et al., 2021). The myosin heavy chain (MHC) composition of single fibers was determined by SDS polyacrylamide gel electrophoresis (SDS‐PAGE). Proteins were separated using a 7% separating gel consisting of 2 M tris HCl (pH 8.6), 50% glycerol, 10% SDS, and 40% (w/v) acrylamide and N,N′‐methylenebis‐acrylamide with a monomer to crosslinker ratio of 37.5:1. The stacking gel consisted of 500 mM tris HCl (pH 6.7), 10% SDS, and 40% (w/v) acrylamide and N,N′‐methylenebis‐acrylamide with a monomer to crosslinker ratio of 37.5:1. Gels were incubated at 4°C and run at a constant voltage of 50 V for approximately 40 h. Fiber types (MHCs I and II; note IIa and IIx were not differentiated for this analysis) were determined by comparing to a commercial standard protein ladder (Bio‐Rad Protein Plus Standard 10–250 kD) of known molecular weights as well as a homogenate cocktail made from rodent muscles with known fiber‐type distributions (soleus and extensor digitorum longus [1 μg protein/4 μl buffer]).

### 2.8 Analysis

To determine the force‐pCa relationship, 7 pCa levels were used: 7.0, 6.6, 6.4, 6.2, 5.7, 5.5, and 4.5. The order of activation was randomized across pCa values. All force values were normalized to CSA. To determine the Ca^2+^ sensitivity of each single fiber, *P*_o_ at each pCa level was normalized to the maximal force elicited by that fiber and the data were fit using a modified Hill equation programmed in software (MATLAB and PRISM 9), where *P*/*P*_o_ is the relative force at a given pCa value and h is the Hill coefficient:

$$\frac{P}{P_{o}}=\frac{1}{{\left(1+10\right)}^{\left(-h\cdot \left(\mathrm{pCa}50-\mathrm{pCa}\right)\right)}}.$$

The pCa value at which 50% of maximal force was elicited (pCa_50_) was used to determine differences in Ca^2+^ sensitivity. Control and trained group force‐pCa curves and pCa_50_ values were compared and further analyzed for a fiber type and training status interaction.

*k*_tr_ was calculated by fitting the force redevelopment curve following rapid shortening and re‐stretch of the fiber to the mono‐exponential equation, *y* = *a* (1 − e^−*kt*^) + *b*. Instantaneous stiffness was calculated as the difference between peak force during the stretch and the average force over the 500 ms prior to the stretch, divided by the change in fiber length induced by the stretch. *k*_tr_ and instantaneous stiffness were compared between control and trained groups and further analyzed for a fiber type and training status interaction.

### 2.9 Statistical analysis

For all statistical analyses, single muscle fibers were treated as independent samples, with 2–8 fibers sampled from each rat. To determine differences in mechanical measures (absolute force, CSA, normalized force, *k*_tr_, instantaneous stiffness, and pCa_50_), a two‐way ANOVA was used with training group and fiber type as between‐group factors. To determine differences in normalized force across pCa, a three‐way ANOVA with a Bonferroni correction was used, with training group and fiber type as between‐group factors and pCa level as the within group factor. Significance was set at an alpha of 0.05 and all statistical analyses were completed with SigmaStat (Systat Software, Inc.; Version 4.0). All data in figures are presented as mean ± SEM.

## 3 RESULTS

### 3.1 Fiber type composition

Of the 63 fibers analyzed, 15 (~24%) were Type I and 48 (~76%) were Type II. Fiber‐type composition was relatively consistent within groups as well, with 6 (~19%) Type I fibers and 25 (~81%) Type II fibers in the control group, and 9 (~28%) Type I fibers and 23 (~72%) Type II fibers in the trained group.

### 3.2 Muscle size and strength

**FIGURE 1:**
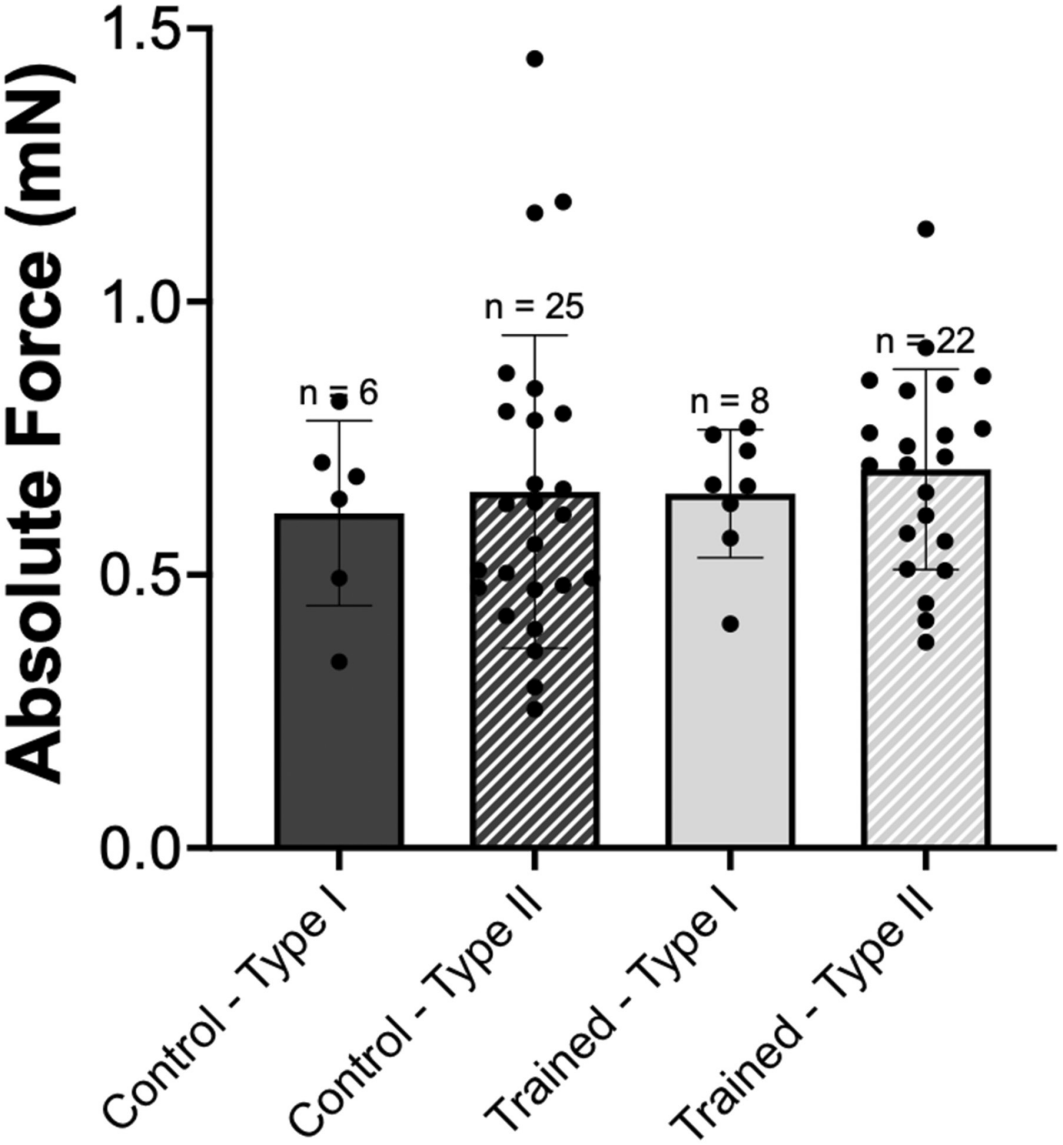
Single muscle fiber absolute force. Comparison of highest single fiber 500 ms moving average plateau force (mN) reached across all pCa levels produced by single fibers from control (dark gray) versus trained (light gray) rats. Each group was further divided into type I (solid) and type II (hashed) fibers. *n* = 2 fibers not included because >3 SD away from the mean. Data are reported as mean ± standard error. No significant differences (*p* > 0.05) were found between groups or fiber types.

**FIGURE 2:**
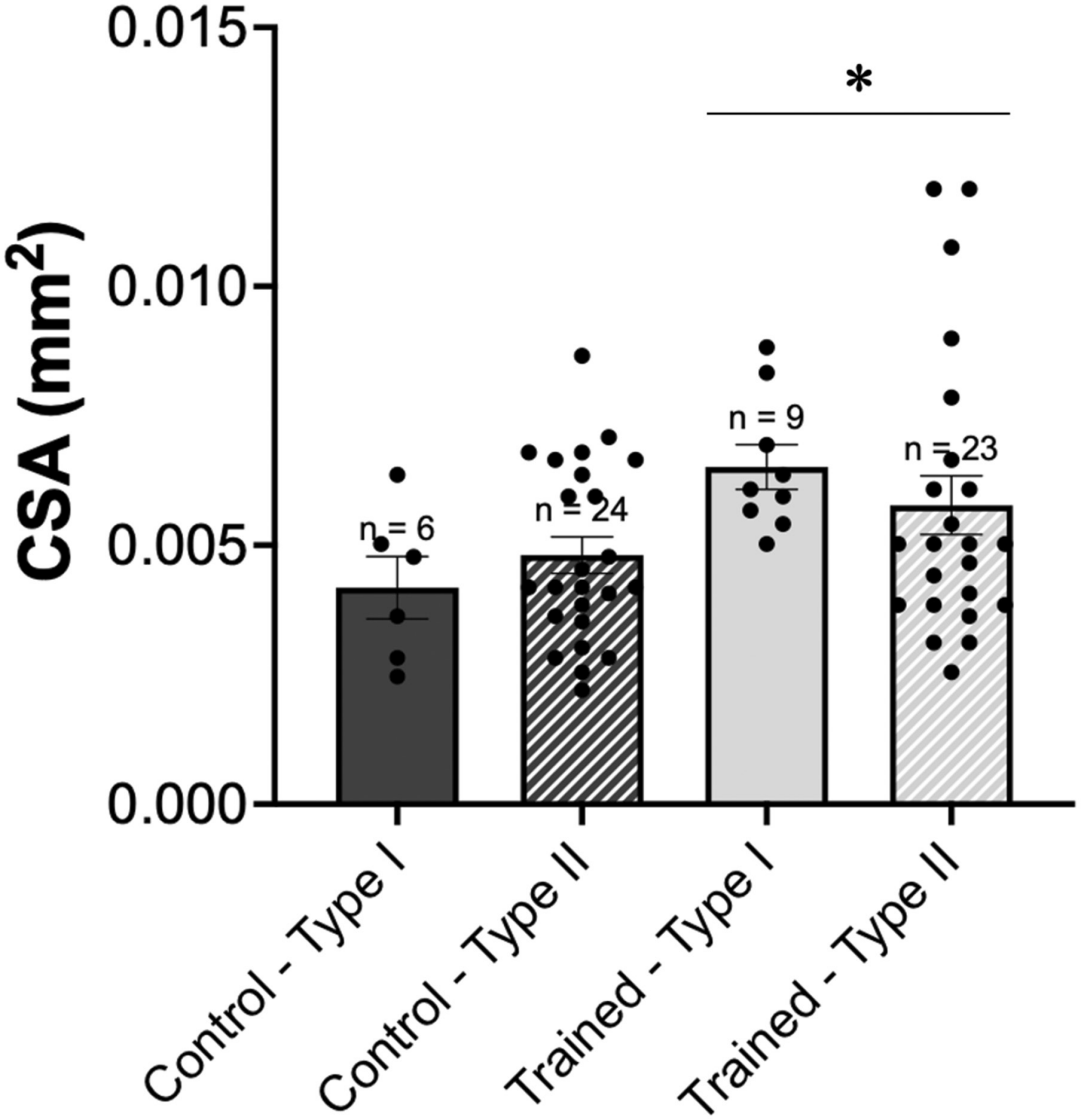
Single muscle fiber cross‐sectional area. Comparison of single fiber CSA (mm^2^) from control (dark gray) versus trained (light gray) rats, further divided into type I (solid) and type II (hashed) fibers. *n* = 1 fibers not included because >3 SD away from the mean. Data are reported as mean ± standard error. *Significantly different from control, *p* < 0.05. No significant differences (*p* > 0.05) were found between fiber types. CSA, cross‐sectional area.

**FIGURE 3:**
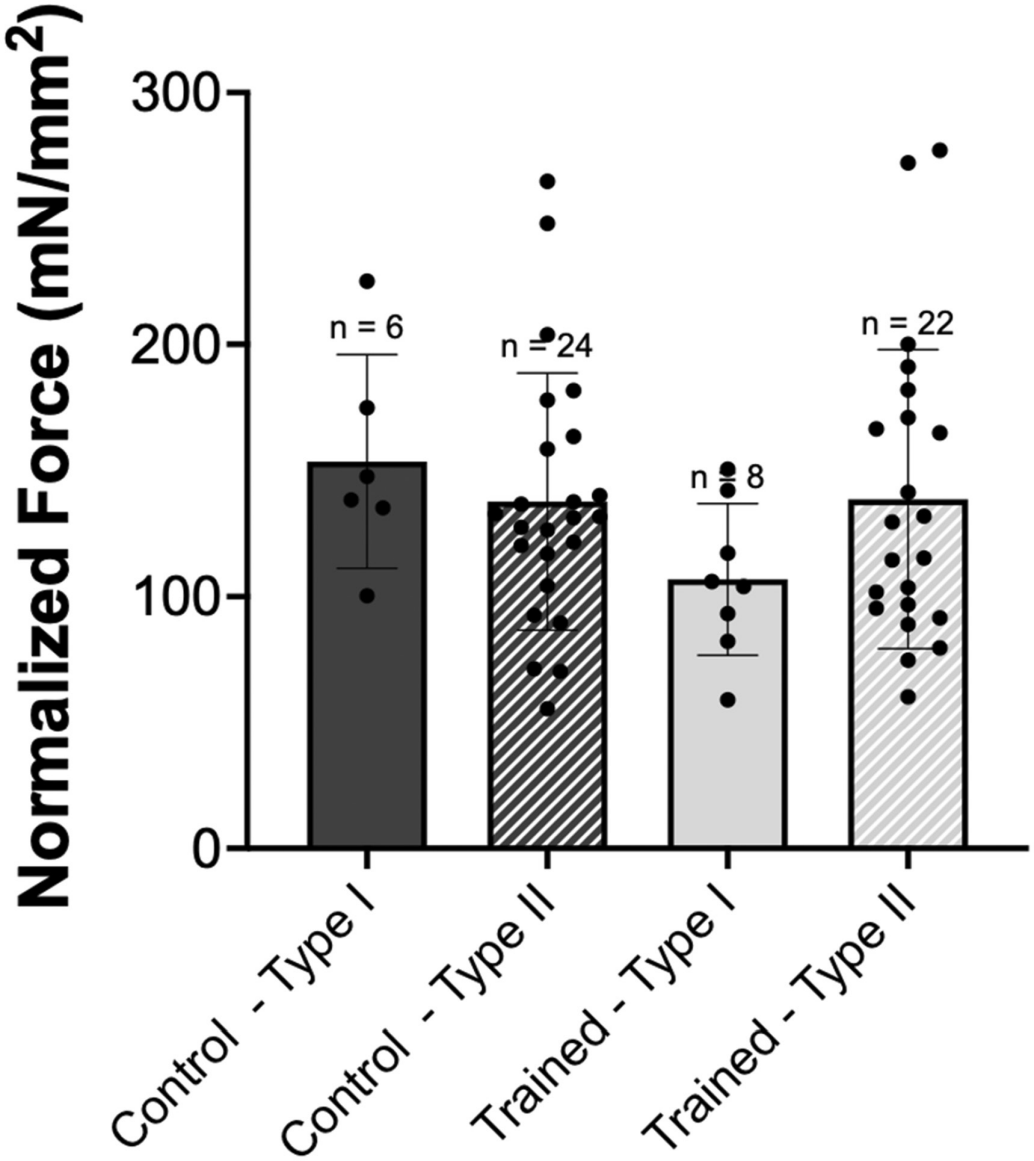
Single muscle fiber normalized force. Comparison of highest single fiber 500 ms moving average plateau force normalized to cross‐sectional area (mN/mm^2^) from single fibers from control (dark gray) versus trained (light gray) rats. Each group was further divided into type I (solid) and type II (hashed) fibers. *n* = 3 fibers not included because >3 SD away from the mean. Data are reported as mean ± standard error. No significant differences (*p* > 0.05) were found between groups or fiber types.

For absolute force, there was no interaction of group × fiber type (*p* > 0.05), nor main effects of group (*p* > 0.05), or fiber type (*p* > 0.05). Therefore, there was no difference in absolute active force between vastus intermedius single muscle fibers from control and trained rats or across fiber type (Figure 1). For single fiber CSA, there was no interaction of group × fiber type (*p* > 0.05), nor main effects of fiber type (*p* > 0.05). However, there was an effect of group (*p* < 0.05) with trained rats having on average 28% larger single fibers (Figure 2). When force was normalized to CSA to account for any differences in myofibrillar protein content, there was no interaction of group × fiber type (*p* > 0.05), nor main effects of group (*p* > 0.05), or fiber type (*p* > 0.05). This indicates that there was no difference in normalized force between groups or fiber types (Figure 3).

### 3.3 Cross‐bridge kinetics and instantaneous stiffness

**FIGURE 4:**
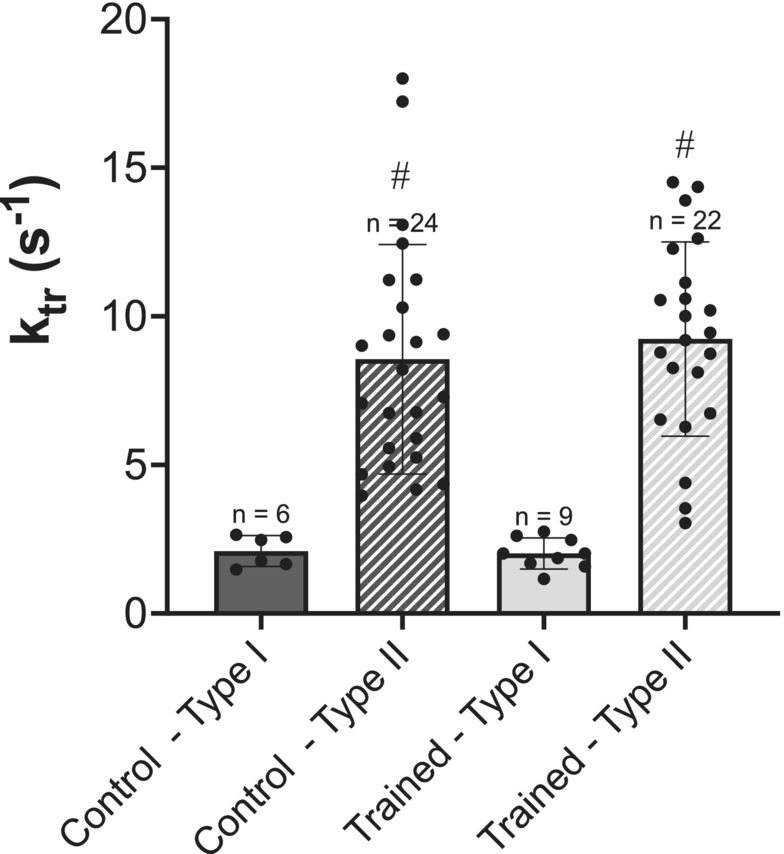
Rate of force redevelopment. Comparison of rate of force redevelopment (*k*_tr_; s^−1^) of single fibers from control (dark gray) versus trained (light gray) rats, further divided into type I (solid) and type II (hashed) fibers. *n* = 2 fibers not included because >3 SD away from the mean. Data are reported as mean ± standard error. ^#^Significantly different from type I fibers, *p* < 0.001. No significant differences (*p* > 0.05) were found between groups.

**FIGURE 5:**
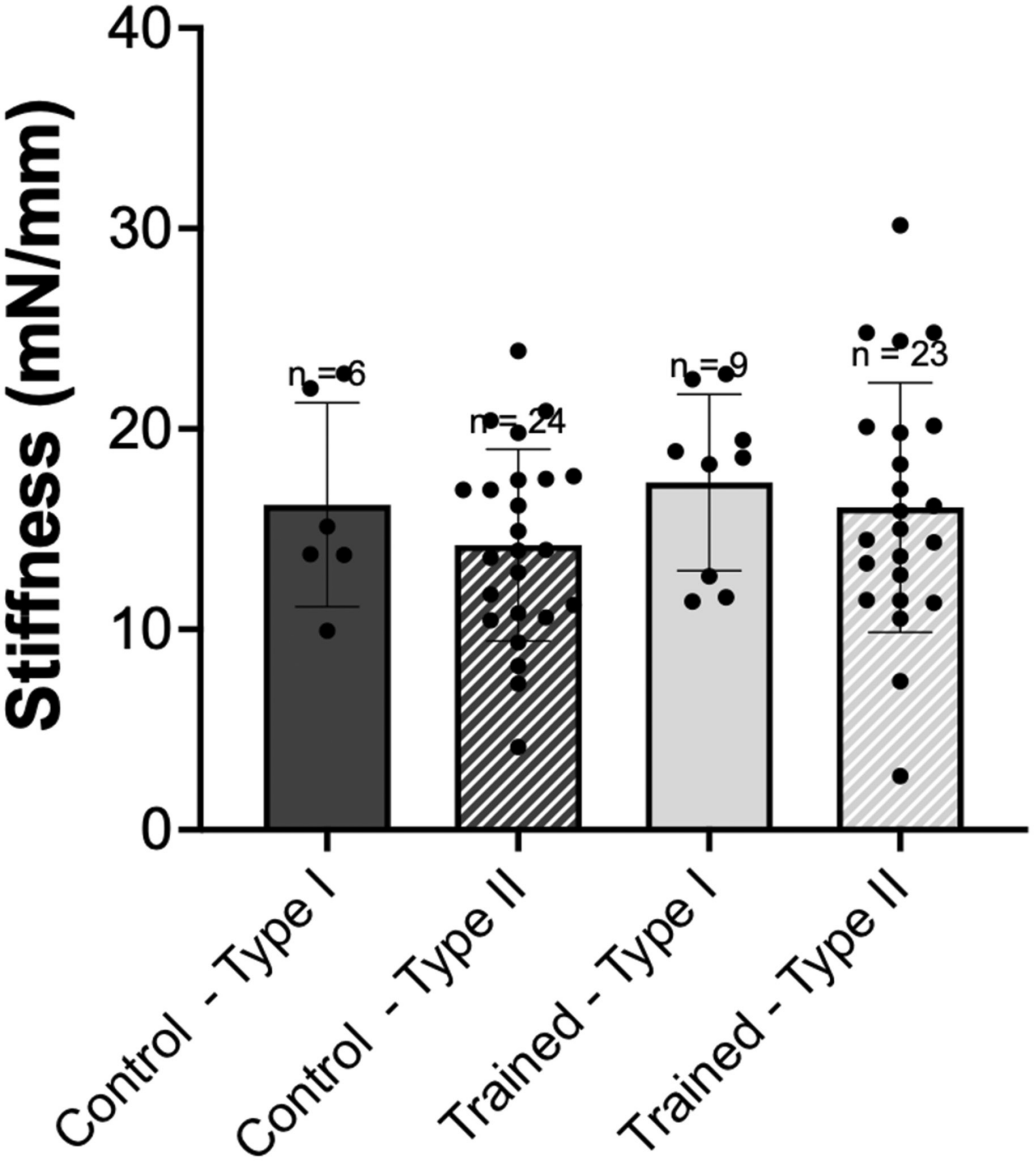
Active instantaneous stiffness. Comparison of stiffness (mN/mm) of single fibers from control (dark gray) versus trained (light gray) rats, further divided into type I (solid) and type II (hashed) fibers. *n* = 1 fiber not included because >3 SD away from the mean. Data are reported as mean ± standard error. No significant differences (*p* > 0.05) were found between groups or fiber types.

For single fiber *k*_tr_, there was no interaction of group × fiber type (*p* > 0.05), nor main effects of group (*p* > 0.05). However, as expected, there was an effect of fiber type (*p* < 0.001) with Type II fibers producing on average 330% faster force development as compared to Type I fibers (Figure 4). For instantaneous active stiffness, there was no interaction of group × fiber type (*p* > 0.05), nor main effects of group (*p* > 0.05), or fiber type (*p* > 0.05). Therefore, there was no difference in active stiffness between vastus intermedius single muscle fibers from control and trained rats or across fiber type (Figure 5). This would indicate there was no modification to the proportion of attached cross‐bridges following downhill running training (i.e., eccentric training).

### 3.4 Calcium sensitivity

**FIGURE 6:**
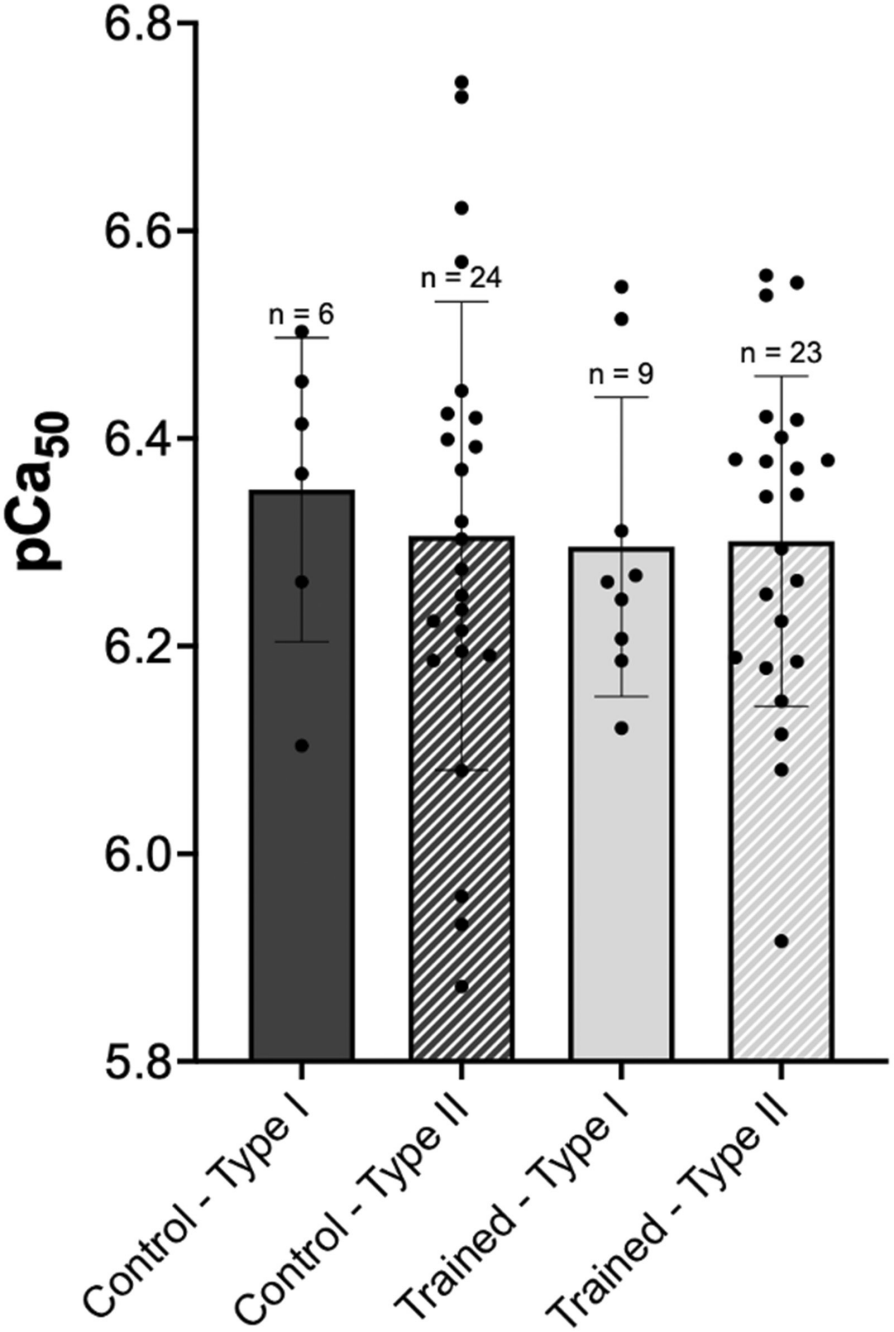
Calcium sensitivity. Comparison of pCa values at which 50% of maximal force was elicited (pCa_50_) between single fibers from control (dark gray) and trained (light gray) rats, further divided into type I (solid) and type II (hashed) fibers. *n* = 1 fibers not included because >3 SD away from the mean. Data are reported as mean ± standard error. No significant differences (*p* > 0.05) were found between groups or fiber types.

**FIGURE 7:**
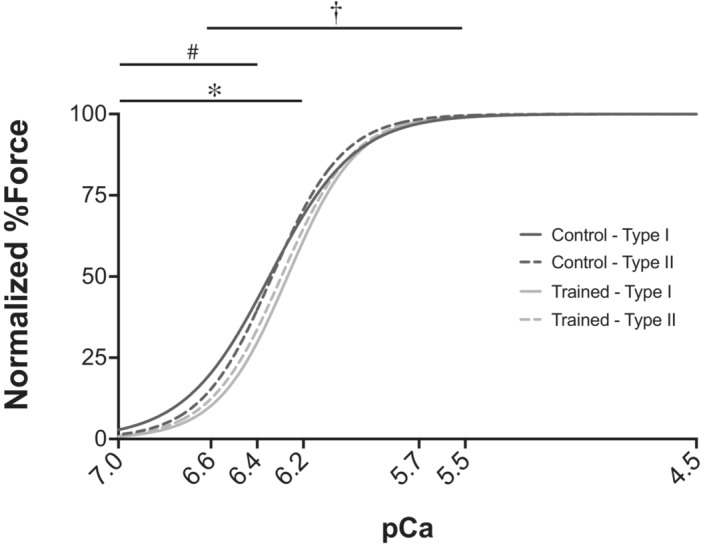
Force‐pCa curves. Normalized force (%P_O_) across pCa levels (7.0, 6.6, 6.4, 6.2, 5.7, 5.5, and 4.5) from control type I (solid dark gray), control type II (broken dark gray), trained type I (solid light gray), and trained type II (broken light gray) fibers. *Significantly different from control *p* < 0.05. ^#^Interaction (group × fiber type). ^†^Significant difference across pCa values.

While visually there appeared to be greater Ca^2+^ sensitivity in Type I as compared with Type II fibers, for pCa_50_ there was no interaction of group × fiber type (*p* > 0.05), nor was there any effect of training group (*p* > 0.05), or fiber type (*p* > 0.05; Figure 6). When considering normalized force across pCa values (Figure 7), there was an interaction of training group × fiber type (*p* = 0.002), such that Type I fibers from the control group produced greater force than training Type I fibers at lower Ca^2+^ concentrations ([Ca^2+^] < 6.4), with no significant differences across groups for Type II fibers. There was an effect of group with control fibers producing greater forces at lower pCa ([Ca^2+^] < 6.2, *p* < 0.05) as compared with the training group. Additionally, there was the obvious expected effect of pCa on force, with increasing force with increasing [Ca^2+^].

## 4 DISCUSSION

This study aimed to assess rat vastus intermedius single muscle fiber adaptations following 4 weeks of eccentric training (i.e., progressively overloaded weighted downhill running). Specifically, force production across [Ca^2+^], CSA, normalized force, *k*_tr_, active instantaneous stiffness, and Ca^2+^ sensitivity were assessed in single muscle fibers from trained and untrained rats. The main findings of the present study were no effects of downhill running on any of the reported mechanical measures that would indicate an increase in Ca^2+^ sensitivity nor any enhancements in muscle function at the single fiber level following eccentric training.

### 4.1 Fiber‐type distribution

The vastus intermedius muscle is generally accepted as a mixed‐type muscle. Previous studies analyzing rat hindlimb fiber types have found that the vastus intermedius muscle is composed of approximately 60% Type II fibers and 40% Type I fibers (Armstrong & Phelps, 1984). However, in the present study, fiber‐type analysis reported closer to 75% Type II fibers and 25% Type I fibers. This raised the possibility of a change in fiber‐type expression caused by training, but this explanation is unlikely as previous studies have determined that 4 week eccentric training protocols did not affect quadriceps muscle MHC isoform distribution (Raue et al., 2005). The greater Type II to Type I fiber ratio could also be due to selection bias of visually viable fibers during dissection and mounting to the muscle testing rig. Furthermore, the fiber‐type ratio of the trained group was not significantly different from that of the control group, indicating that an effect of training was unlikely.

### 4.2 Effect of downhill running training on muscle size and strength

There was no effect of downhill running training on absolute or normalized single muscle fiber force production as compared to the control group (Figures 1 and 3). This would indicate that previously observed improvements in muscle function following eccentric training (Douglas et al., 2017; Franchi et al., 2014) are not necessarily owing to adaptations in mechanical function at the cellular level. With respect to muscle fiber size, single fibers from trained rats had on average 28% larger CSAs than control rats, independent of fiber type. However, the absence of a concomitant increase in force indicates that this training adaptation was not functionally relevant and could be related to fiber swelling caused by shifts in water and ion flow during exercise (Monti et al., 2021; Sjogaard et al., 1985; Watanabe et al., 2019), or variable swelling of skinned fibers during testing (Kalakoutis et al., 2021).

Previous studies have yielded inconsistent results regarding muscle size and strength adaptations following eccentric training and appear to be dependent on the animal model, muscle, and training protocol used. Following 14 weeks of downhill training in rats, CSA of Type II fibers from the EDL was greater than controls, with no difference observed across groups for Type I fibers (Lynch et al., 1997). Additionally, there was no effect of training on CSA of fibers from the soleus, regardless of fiber type. Accordingly, eccentric training has been shown to produce preferential increases in the size of Type II fibers (Douglas et al., 2017); however, in the present study, in the training group there was an increased CSA for both Type I and II fibers (Figure 2). Recently, Hinks et al. (2022) reported no differences in muscle size or isometric force production for the soleus muscle tested in vitro following 4 weeks of downhill running between the control and training groups. Of note, this is the same sample of rats used for single fiber analysis in the present study. With regard to muscle strength, our findings seem to be consistent with the lack of single muscle fiber performance enhancement reported previously following downhill running training (Mashouri et al., 2021). Contrary to the absence of performance enhancements observed by Mashouri et al. (2021) and the present study, a single bout of downhill running followed by a 4‐week sedentary or downhill running training regimen revealed no group differences in muscle fiber CSA, but an increase in relative hindlimb grip force was observed in the trained group relative to the sedentary group (Zou et al., 2015). It should be noted that this assessment of strength was completed in vivo at the level of the whole muscle, which may provide a reason for its divergence from previous data as well as the findings of the present study. However, it is interesting that performance enhancements may have occurred in the absence of changes in size while in the present study, changes in CSA occurred without strength enhancements.

### 4.3 Effect of downhill running training on mechanical function

There was no effect of downhill running training on *k*_tr_ or active instantaneous stiffness (Figures 4 and 5), indicating that neither the rate of transition of non‐force bearing to force bearing cross‐ bridge states nor the proportion of weakly‐to‐strongly bound cross‐bridge states were altered with eccentric training. It has previously been shown that *k*_tr_ increases sigmoidally with increases in [Ca^2+^] with the greatest values being reported at pCa 4.5 (Metzger & Moss, 1991; Metzger et al., 1989). Additionally, in human single vastus lateralis (VL) muscle fibers, *k*_tr_ and instantaneous stiffness have both been shown to be influenced by Ca^2+^ sensitivity (Mazara et al., 2021). As such, we expected that any effects of eccentric training on Ca^2+^ sensitivity would be accompanied by changes in *k*_tr_. Therefore, the absence of an effect of group on either measure of mechanical function in the present study may simply be due to a lack of effect of eccentric training on Ca^2+^ sensitivity. As expected, *k*_tr_ was significantly faster in Type II fibers as compared to Type I fibers regardless of training group (Figure 4). This effect has been observed previously in chemically permeabilized single muscle fibers with the rate of cross‐bridge attachment and force generation being markedly greater in Type II fibers (Metzger & Moss, 1990). These differences are thought to be directly related to different rates of cross‐bridge cycling between myosin isoforms and greater cooperativity of Ca^2+^ activation in Type II compared to Type I fibers (Wahr et al., 1997). Our findings were also consistent with previous work from our lab which showed faster *k*_tr_ in Type II fibers as compared with Type I at maximal activation (Mazara et al., 2021).

### 4.4 Effect of downhill running training on calcium sensitivity

The pCa value at which 50% of maximal force was elicited (pCa_50_) did not differ between fibers from the training and the control group, regardless of fiber type (Figure 6), suggesting that eccentric training did not alter Ca^2+^ sensitivity. However, analysis of the normalized force‐pCa curve revealed that Ca^2+^ sensitivity at low Ca^2+^ concentrations may have been reduced by training, particularly in Type I fibers (Figure 7). The observation that pCa_50_ remained unaltered by training agrees with previous studies comparing trained swimmers with control subjects (Fitts et al., 1989), and masters runners with age‐matched controls (Widrick et al., 1996). Furthermore, our finding that control Type I fibers produced greater forces than trained Type I fibers at low Ca^2+^ concentrations is consistent with reports of decreased Type I fiber Ca^2+^ sensitivity following high intensity interval training (Lamboley et al., 2020). As such, we can infer that aerobic‐based training may not be sufficient to cause changes in Ca^2+^ sensitivity, and interval training can have a detrimental effect on Ca^2+^ sensitivity. Accordingly, increases in Ca^2+^ sensitivity have been found in previous training studies with greater resistance training components, including one investigating the effect of 8 weeks of plyometric (stretch/shortening) training on Ca^2+^ sensitivity in human VL single muscle fibers (Malisoux et al., 2006). Their study reported improved performance outcomes post‐training and found that Ca^2+^ sensitivity was enhanced in Type I fibers but not significantly different in Type II fibers (Malisoux et al., 2006). It was speculated that alterations in Ca^2+^ sensitivity may be owing to alterations in the expression of slow or fast troponin T (TnT) isoforms; however, it was reported that slow TnT isoforms were associated with Type I fibers and fast TnT isoforms were associated with Type II fibers, regardless of training (Malisoux et al., 2006). Additionally, a 12‐week progressive resistance training program in older women yielded similar fiber type‐dependent results with increases in Ca^2+^ sensitivity of Type I but not Type II fibers. The authors suggested that increased cooperative binding or altered cross‐bridge cycling played a role in the observed fiber type‐specific changes in Ca^2+^ sensitivity (Godard et al., 2002). It is interesting to note that the training protocols in these studies lasted 8 and 12 weeks, respectively, which are significantly longer than the duration of our training intervention. As such, it is also possible that 4 weeks of eccentric training is simply not long enough for performance enhancements to occur.

Plyometric training (Malisoux et al., 2006), progressive resistance training (Godard et al., 2002), and high intensity interval training (Lamboley et al., 2020) in humans, and ‐ as shown in the present study ‐ downhill running training in rats all preferentially affected Ca^2+^ sensitivity of Type I fibers, albeit in different directions. This raises the possibility that Type II fibers are less prone to alterations in Ca^2+^ sensitivity, a concept that may be supported by the fact that Type I muscle fibers have previously been shown to have higher Ca^2+^ sensitivities (Danieli‐Betto et al., 1990; Lamboley et al., 2015; Ruff, 1989) and may therefore be more readily altered by interventions. Previous studies showing that muscle unloading leads to decreases in Ca^2+^ sensitivity, particularly in Type I fibers, credited changes in the expression of muscle regulatory or contractile proteins, or the spacing between filaments of the myofibrillar lattice (McDonald & Fitts, 1995; Widrick et al., 1998). Alternatively, with respect to eccentric training, the preferential effect on Type I fibers may instead be related to the aerobic nature of the downhill running training protocols that selectively recruit slow‐twitch muscle fibers (Wakeling et al., 2006). One study reported an increase in Ca^2+^ sensitivity in rat Type II muscle fibers following an acute bout of running (Xu et al., 2018), and several groups have reported training‐induced transient increases in Type II muscle fiber Ca^2+^ sensitivity owing to a reversible oxidation‐related process (Lamboley et al., 2020; Xu et al., 2018), but we did not observe any increases in Ca^2+^ sensitivity in Type II fibers. We expect that this was the case because our rats rested for 72 h following their last bout of training prior to tissue collection and increases in Type II fiber Ca^2+^ sensitivity have been shown to almost completely disappear after 24 h of rest (Xu et al., 2018). Given that these Type II fiber‐specific changes tend to be short‐term, it would stand to reason that the chronic training component of the present study is what provides a stimulus specific to Type I fibers. However, despite observing a preferential effect on Type I fibers, we cannot identify the precise underlying mechanism of fiber type‐specific changes in Ca^2+^ sensitivity.

It is also important to note that the observed decrease in Ca^2+^ sensitivity in the eccentrically trained group in the present study was only significant at low [Ca^2+^] (pCa >6.2). This indicates that while eccentric training likely had no effect on Ca^2+^ sensitivity at maximal activation, there may have been a negative effect of training on submaximal contractions. This would explain why differences were observable in the force‐pCa curves but not significant in pCa_50_ values, as pCa_50_ was between 6.2 and 6.4 for all groups and fiber types. It is also possible that it was simply the main effect of training to increase CSA without concomitant increases in force that resulted in lower normalized forces. This difference may not have been significant at higher [Ca^2+^] but become more extreme between pCa 6.2 and 7.0.

## 5 CONCLUSION

Despite reports of improved mechanical function following eccentric training at the whole muscle and joint levels, it does not appear that cellular mechanical mechanisms of muscle contractility are contributing to these performance enhancements, at least with this training protocol in the rat vastus intermedius, as single muscle fiber mechanical function and Ca^2+^ sensitivity were not altered following training.

## AUTHOR CONTRIBUTIONS

Emma F. Hubbard, Avery Hinks, Parastoo Mashouri, and Geoffrey A. Power conceived and designed research and performed experiments. Emma F. Hubbard and Geoffrey A. Power analyzed data, interpreted results, prepared figures, and drafted manuscript. Emma F. Hubbard, Avery Hinks, Parastoo Mashouri, and Geoffrey A. Power edited and revised manuscript and approved final version of the manuscript. All authors have read and approved the final version of the manuscript and agree with the order of presentation of the authors.

## FUNDING INFORMATION

This project was supported by the Natural Sciences and Engineering Research Council of Canada (NSERC).

## CONFLICT OF INTEREST

The authors declare that they have no competing interests.

## ETHICS STATEMENT

All procedures were approved by the Animal Care Committee of the University of Guelph.
